# Direct Analysis of hCGβcf Glycosylation in Normal and Aberrant Pregnancy by Matrix-Assisted Laser Desorption/Ionization Time-of-Flight Mass Spectrometry

**DOI:** 10.3390/ijms150610067

**Published:** 2014-06-05

**Authors:** Ray K. Iles, Laurence A. Cole, Stephen A. Butler

**Affiliations:** 1 Williamson Laboratory for Molecular Oncology, St Bartholomews Hospital, London EC1A 7BE, UK; E-Mail: Butlersa1@googlemail.com; 2 ELK Foundation for Health Research, An Scoil Monzaird, Crieff PH7 4JT, UK; 3 MAP Diagnostics Ltd., Ely, Cambridgeshire CB6 3FQ, UK; 4 USA hCG Reference Service, Angel Fire, NM 87710, USA; E-Mail: larry@hcglab.com

**Keywords:** hCG, hCGβcf, MALDI TOF MS, pregnancy, hydatidiform mole, hyperemesis gravidarum, glycosylation

## Abstract

The analysis of human chorionic gonadotropin (hCG) in clinical chemistry laboratories by specific immunoassay is well established. However, changes in glycosylation are not as easily assayed and yet alterations in hCG glycosylation is associated with abnormal pregnancy. hCGβ-core fragment (hCGβcf) was isolated from the urine of women, pregnant with normal, molar and hyperemesis gravidarum pregnancies. Each sample was subjected to matrix-assisted laser desorption/ionization time-of-flight mass spectrometry (MALDI TOF MS) analysis following dithiothreitol (DTT) reduction and fingerprint spectra of peptide hCGβ 6–40 were analyzed. Samples were variably glycosylated, where most structures were small, core and largely mono-antennary. Larger single bi-antennary and mixtures of larger mono-antennary and bi-antennary moieties were also observed in some samples. Larger glycoforms were more abundant in the abnormal pregnancies and tri-antennary carbohydrate moieties were only observed in the samples from molar and hyperemesis gravidarum pregnancies. Given that such spectral profiling differences may be characteristic, development of small sample preparation for mass spectral analysis of hCG may lead to a simpler and faster approach to glycostructural analysis and potentially a novel clinical diagnostic test.

## 1. Introduction

In a post-genomic era the importance of proteoforms has come to the fore [1], and it is the subtleties of the proteoform that underlay many pathologies not yet characterized at a genetic level. This is not simply splice variants but the form a protein takes within a functional cellular and physiological system. Critical to clinical functionality of a coded protein are its post translational modifications, e.g., pre- and pro-peptide cleavage, phosphorylation and glycosylation. Detection and relative quantification of particular proteoforms will form the bases of new biomarker discovery and not necessarily simple measurement of any given mass of protein [2].

The detection of human chorionic gonadotropin (hCG) is used extensively in obstetrics and gynecology for the detection and monitoring of pregnancy. The hormone is an αβ hetero-dimeric glycoprotein with eight glycosylation sites, comprising four *N*-linked oligosaccharides and four *O*-linked oligosaccharides. Two *N*-linked oligosaccharides are attached to each of the subunit polypeptide chains by β-*N*-glycosidic bonds to asparagine residues. These moieties share the same basic structural characteristics: *N*-acetylglucosamine (GlcNAc) is attached to an asparagine residue followed by another GlcNAc, mannose, and two more branches of mannose. This is the mono-antennary pentasaccharide core with the remaining components being variable [3,4,5]. The *O*-linked oligosaccharides are attached by α-*O*-glycosidic bonds onto serine residues of the β-subunit carboxyl terminal peptide [6,7,8,9].

Carbohydrate heterogeneity has been extensively reported for the free β-subunit of hCG (hCGβ) with variable mono-, bi-, and tri-antennary carbohydrate structures being found in normal and abnormal pregnancies, as well as in gestational trophoblastic disease and in particular choriocarcinoma and early pregnancy [10,11,12,13,14]. In general, a greater proportion of tri-antennary oligosaccharide structures are usually indicative of abnormalities in pregnancy, while bi-antennary forms account for the majority of structures found in normal pregnancy [13].

hCG is excreted intact into the urine, as documented by extensive implementation of urinary pregnancy testing. However, hCG is also degraded in liver and kidneys and a large proportion of immunoreactive hCG in the urine is attributed to this urinary degradation product of the hCGβ subunit hCG β-core fragment (hCGβcf). The carbohydrate structures of the hCGβcf have been studied independently [15,16] and the molecule is composed of peptides, β 6–40 and β 55–92, connected by four disulfide bridges. It retains many of the antigenic determinants of the original hCGβ molecule prior to metabolism, which occurs primarily in the kidney [17]. The β 6–40 polypeptide chain contains the two hCGβ *N*-linked carbohydrate moieties, although the oligosaccharides are truncated due to metabolism. Urinary hCGβcf can be isolated with relatively straightforward procedures [15] from a simple urine sample and offers a convenient way of providing insights into glycosylation of the hCGβ subunit and therefore the hCG from which it was derived [18]. This presents an opportunity to indirectly study pregnancy disorders known to exhibit glycoform variants of hCG.

Matrix-assisted laser desorption/ionization time-of-flight mass spectrometry (MALDI TOF MS) is a technique that can be used for the determination of the mass of macromolecules, originally developed by Karas & Hillenkamp [19]. MALDI TOF MS can be used in the characterization of glycopeptides [20] and/or oligosaccharides that are released from glycoproteins with the use of enzymatic digestion [21,22,23]. Dithiothreitol (DTT) can also be used in situations where disulfide linkages are present and can reduce the mass of peptides bringing them into relatively optimum resolution for this mass spectrometer.

In the case of hCGβcf, the amino acids β 55–92 are linked to β 6–40 from the original β-subunit in hCG. After disulfide reduction, these two peptides along with glycosylation moieties can be analyzed by MALDI TOF MS and oligosaccharide masses calculated by subtraction of the peptide mass of the β 6–40 chain from the observed peak mass of each glycoform. Carbohydrate heterogeneity has been reported on hCGβcf and a population of mono- and bi-antennary structures has been proposed by various studies [24,25,26,27]. Using a MALDI TOF MS technique we have previously shown that the remaining oligosaccharide structures found on hCGβcf do not possess sialic acid and the extent to which those structures are truncated prior to urinary excretion as hCGβcf [28]. This made it possible to analyze glycosylation moieties whilst still attached to the peptide, thus eliminating the need for glycosidase digestion. However, this previous work was conducted on a pooled sample preparation and there has, as yet, been no report of hCGβcf glycosylation patterns from individual patients. In order to provide some insights in hCGβcf glycosylation in aberrant pregnancies, we used the same MALDI TOF MS technique to analyze hCGβcf isolated from individual patient samples with normal pregnancy or conditions such as molar pregnancy and hyperemesis gravidarum.

## 2. Results and Discussion

### 2.1. Mass Spectral Profiles

**Figure 1:**
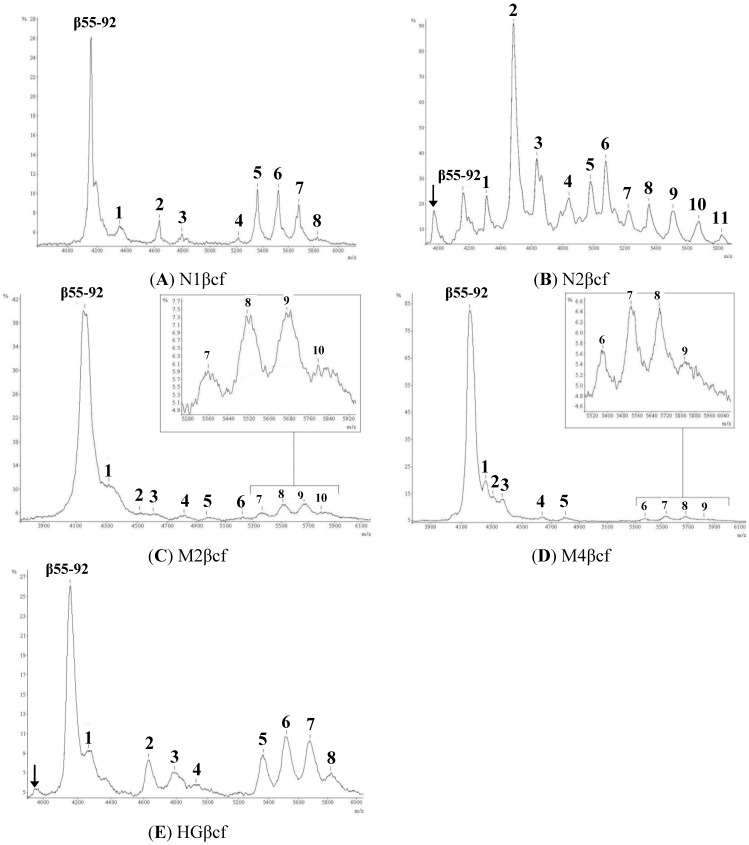
Matrix-assisted laser desorption/ionization time-of-flight mass spectrometry (MALDI TOF MS) of human chorionic gonadotropin β-core fragment (hCGβcf) treated with dithiothreitol (DTT). hCGβcf purified from pregnancy urine samples; Normal (**A**,**B**), Molar (**C**,**D**) and Hyperemesis Gravidarium (**E**). Disulfide linkages were reduced using DTT. The indicated peak at *m*/*z* 4156.8 (β 55–92) appears in all samples and represents the unglycosylated peptide of beta-core. Arrowed peak (↓) only appears in samples N2βcf and HGβcf and indicates a fragment smaller than β 55–92 and as such is likely to be β 6–40 with minimal or no-glycosylation. All remaining peaks are attributed β 6–40 glycopeptides and described in Table 1.

**Figure 2:**
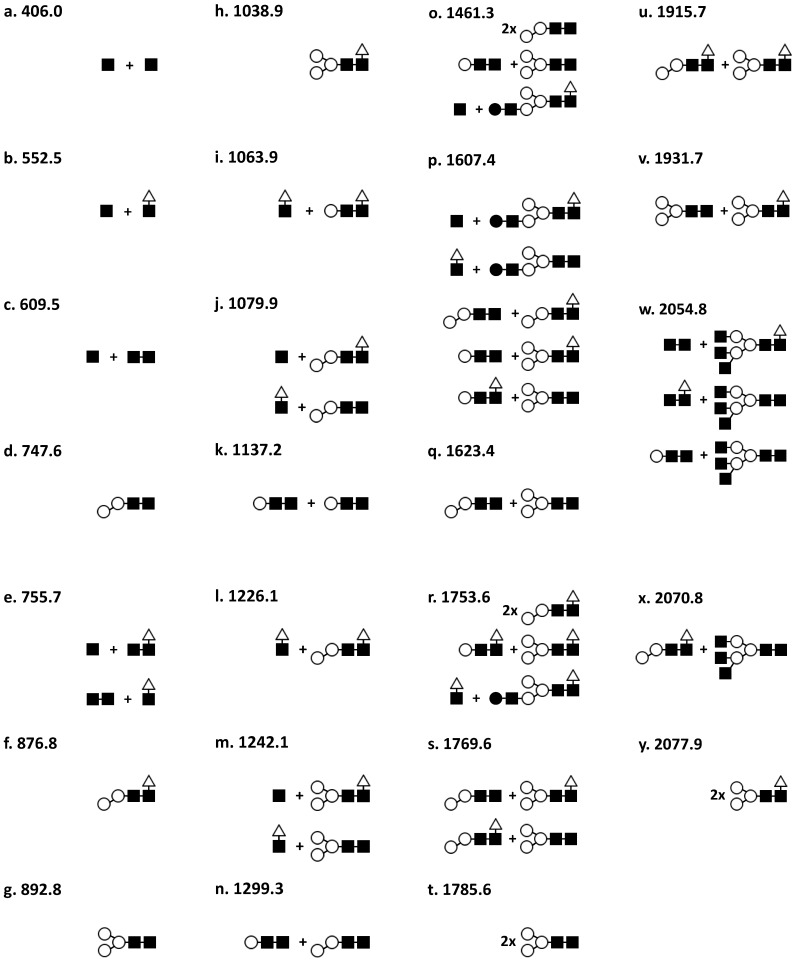
Oligosaccharide structures of hCGβcf. Structures identified in samples used in this study. The information for each structure includes; structure letter, schematic and molecular weight (Da). ■, GlcNAc (221.2 Da); ○, mannose (180.2 Da); ∆, Fucose (164.2 Da); ●, Galactose (180.2 Da).

hCGβcf purified from pregnancy urine samples (normal, molar and hyperemesis gravidarium) subjected to MALDI TOF MS generated mass spectra for hCGβcf displaying a broad peak between *m*/*z* 8700 and 10,700, as published previously [28]. On reduction of the disulfide linkages using DTT, this broad peak was replaced by a set of lower molecular weight peaks (Figure 1). A peak at *m*/*z* 3950 was seen in the spectra from hCGβcf samples N2βcf and HGβcf (Figure 2b,e). Common to all samples was the peak at *m*/*z* 4156.8, corresponding to the non-glycosylated hCGβcf peptide β 55–92 (Figure 1).

### 2.2. Determination of Glycostructures

Prediction of the glyco-structures that resulted in the remaining peaks was achieved by the subtraction of the corresponding mass of the primary amino acid sequence of β 6–40 from the observed *m*/*z* values corresponding to the glycosylated isoforms (Figure 2 and Table 1). Despite the fact that the exact predicted mass of the hCGβcf asparagine-linked carbohydrate moieties were not observed directly, the low percentage errors between the observed and expected mass match of the peaks acquired show that it is likely that these glycoforms were detected. The proposed carbohydrate moieties identified from the mass spectra are shown in Figure 2. Each of the five pregnancy samples contained between 8 and 11 out of the 25 glycosylated forms of β 6–40 identified in this set of samples (Figure 2 and Table 1).

**Table 1:** Identifying MALDI TOF MS peaks. For each peak in each sample; an inferred oligosaccharide (CHO) mass was calculated and best fit structure assigned (Figure 2). The theoretical mass of the glycopeptides (β 6–40 plus CHO moiety) was then calculated as percentage fit (mass match) to the observed peak mass. The calculated relative abundance of each observed peak represents the proportion of area under the curve for the mass spectral range (*m*/*z* 4200–6000) for that peak/glycopeptides (% abundance).

Peak	Observed Mass (*m*/*z*) [M + H]^+^	Predicted Carbohydrate Structure (Figure 2)	Predicted Mass (Da) of Glycopeptide	% Mass Match	% Abundance
	**N1bcf**				
1	4353.8	c	4361.9	0.9982	16.2
2	4634.7	f	4629.2	1.0012	5.1
3	4798.8	h	4790.8	1.0017	4.4
4	5220.8	o	5213.7	1.0014	3.2
5	5366.7	p	5359.8	1.0013	18.1
6	5529.1	s	5522.0	1.0013	22.8
7	5689.8	v	5684.1	1.0010	17.8
8	5840.6	y	5830.3	1.0018	12.4
	**N2bcf**				
1	4307.7	b	4277.9	1.007	4.7
2	4477.6	d	4483.0	1.0012	35.2
3	4630.9	g	4630.9	1.0030	17.3
4	4837.4	j	4832.3	0.9989	6.6
5	4976.6	l	4978.5	1.0004	5.3
6	5074.3	n	5051.4	0.9955	7.5
7	5219.7	o	5213.7	0.9899	3.3
8	5352.6	p	5359.8	1.0013	5.9
9	5504.9	r	5506.0	1.0002	7.3
10	5672.8	u	5568.1	0.9992	5.3
11	5820.9	x	5823.2	1.0004	1.6
	**M2bcf**				
1	4310	b	4304.9	0.9988	24
2	4515.2	e	4508.1	0.9984	0.3
3	4601.7	f	4628.4	1.0058	0.6
4	4805.8	i	4816.3	1.0022	13.1
5	4966.4	m	4994.5	1.0056	9.8
6	5219.1	o	5213.7	0.9970	1.7
7	5359.5	p	5359.8	1.0001	9.8
8	5518.9	s	5522.0	1.0006	13.4
9	5673	u	5668.1	0.9991	15.3
10	5796.1	w	5807.2	1.0019	12
	**M4bcf**				
1	4256.3	a	4158.4	0.9770	25.6
2	4305.2	b	4304.9	0.9999	16.2
3	4369	c	4361.9	0.9984	24.5
4	4639.1	f	4629.2	0.9979	6.1
5	4801.5	h	4791.3	0.9979	8.1
6	5378.9	q	5375.8	0.9994	4.1
7	5535.7	t	5538.0	1.0004	7.3
8	5686.9	v	5684.1	0.9995	5.7
9	5824.9	x	5823.2	0.9997	2.4
	**HGbcf**				
1	4265.4	b	4304.4	1.0091	12.2
2	4628.3	f	4629.2	1.0002	9.2
3	4790.2	h	4791.3	1.0002	8.9
4	4929.4	k	4889.6	0.9919	3.1
5	5361.6	p	5359.8	0.9997	13.5
6	5516.8	s	5522.0	1.0079	21.7
7	5675.7	u	5568.1	0.9987	14.9
8	5820.2	x	5823.2	1.0005	16.5

### 2.3. Relative Abundance of Glycoforms

The most commonly detected glycol-structure found in 4 of 5 of the samples were Figure 2 structures b (*m*/*z* 552.5), f (*m*/*z* 876.8), p (*m*/*z* 1607.4); and in 3 of 5 samples h (*m*/*z* 1038.9), o (*m*/*z* 1461.3), s (*m*/*z* 1769.6) and v (*m*/*z* 1915.7). Collectively structures b (*m*/*z* 552.5), p (*m*/*z* 1607.4), and s (*m*/*z* 1769.6) represent a third of the peak abundance of all the spectra.

The incidence of the remaining glyco-structures was low as was the abundance of the mass spectra generated for the urine samples from normal pregnancies; sample N1βcf had one unique peak at *m*/*z* 2077.9 (carbohydrate structure y in Figure 2) and sample N2βcf four- structures: d (*m*/*z* 747.6), g (*m*/*z* 892.8), j (*m*/*z* 1079.9) and l (*m*/*z* 1226.1). Peaks corresponding to structures e (*m*/*z* 755.7), i (*m*/*z* 1063.9), m (*m*/*z* 1242.1) and w (*m*/*z* 2054.8) were present only in the hCGβcf purified from M2βcf, whilst the spectra for the second molar pregnancy urine sample M4βcf displayed peaks representing structure q (*m*/*z* 1623.4) and t (*m*/*z* 1769.6). Interestingly the hCGβcf preparation from the hyperemesis gravidarium pregnancy urine did not reveal any unique glycoforms.

Fucose at 1–6 of the basal GlcNac was a common retained feature of the residual glycosylation moieties, occurring in 16 of the 25 identified structures and in terms of abundance could be accounted for in 76% of the peak areas of the combined samples.

The glyco-structures that contributed to the greatest proportion of samples are; N1βcf—s (*m*/*z* 1769.6) (22.8%); N2βcf—d (*m*/*z* 747.6) (35.2%), M2βcf—b (*m*/*z* 552.5) (24%), M4βcf—a (*m*/*z* 406.0) (24.5%) and HGβcf—s (*m*/*z* 1769.6) (21.7%). Mono-antennary structures (*m*/*z* 406–1226.1) and bi-antennary structures (*m*/*z* 892.8–2077.9) were found in all samples. Tri-antennary carbohydrate moieties w (*m*/*z* 2054.8) and x (*m*/*z* 2070.8) were only detected in molar pregnancy-M2βcf and Hyperemesis gravidarum-HGβcf samples.

### 2.4. Discussion

HCG is produced by placental trophoblast cells and is a glycoprotein hormone in the diagnosis of pregnancy testing and in the detection of cancer. It would be a significant improvement on current methods to develop a rapid and reliable analytical technique for the characterisation of peptide and carbohydrate portions of hCG rather than a simple quantification of serum or urine levels. By differentiating between those hCG moieties present and with the development of analytical peptide standards for hCG, progress can really be made in identifying hCG glyco-variants as specific clinical diagnostic markers. As such the utilisation of mass spectrometry for the detection and characterisation of hCG would provide an additional diagnostic tool in both the monitoring of pregnancy and cancer.

This study examined the structural heterogeneity of hCGβcf from individuals with normal pregnancy, hydatidiform mole and hyperemesis gravidarum. MALDI TOF MS was used to analyse hCGβcf isolated from individual pregnancy urine samples. Reduction of hCGβcf purified from normal pregnancy urine resulted in the separation of the two peptides; non-glycosylated (β 55–92) and glycosylated (β 6–40) chains corresponding to the mass spectral peaks at *m*/*z* 4156.8 and 5840.6 respectively (See Figure 1). In addition to the peaks attributed to the non-glycosylated and the glycosylated peptide and its glycoforms (discussed extensively below) hCGβcf mass spectra from samples N2βcf and HGβcf displayed a peak at *m*/*z* 3950. This peak was not detected in the pooled urine samples from multiple pregnancies from our previous study. The *m*/*z* value of this species is too high to attribute the peak to a non-glycosylated β 6–40, we speculate therefore that this is a glycoform of β 6–40 with a carbohydrate moiety of approximate molecular mass 197.6 Da. Studies of the biosynthesis of *N*-linked sugar chains have demonstrated that a common core of 3 mannose residues forming two branches of GlcNAc_2_ (Man_3_GlcNAc_2_) is transferred *en bloc* to the polypeptide chain and that the removal of portions of this unit and addition of other sugar residues occur during subsequent processing [29,30]. This processing of the *N*-linked oligosaccharide and also the attachment of the sugars to specific serine or threonine residues takes place in the Golgi apparatus [31]. In the first instance it is possible to suggest that this peak is due solely to the attachment of either galactose or mannose directly on the β 6–40, as there molecular weights are both 180.2 Da. However, in line with the mechanism by which *N*-linked carbohydrates are processed, it may be that this peak represents β 6–40 with one GlcNAc (*m*/*z* 221.21) suggesting that this oligosaccharide may have been removed during processing and modified no further.

The remaining mass spectral peaks are attributed to the multiple glycosylated forms of the peptide β 6–40. Absolute quantification of the relative amounts of each carbohydrate moiety was not possible using this method, one of the perceived restrictions of MALDI TOF MS is its inability to quantitate from spectra. However, we applied a semi-quantitative approach by determining the areas under the peaks of the reduced peptides, similar to that used for data generated by HPLC. These results suggest that hCG is *N*-linked hyperglycosylated to a greater extent in disease and abnormal pregnancy as has been previously described [13,14] and that these glycosylation moiety variation structures are reflected through to the pattern and abundance of urinary metabolite hCGβcf glycoforms. This combined finding suggests a possible use of hCGβcf glycoform analysis by MALDI TOF MS or other methodologies as a novel marker of these diseases.

However, before the use of MALDI TOF MS, as described here, is a clinical reality several technical problems need to be overcome: The first is that we examined purified hCGβcf originating from large pools of collected urine. This volume collection alone renders this approach in-practical for routine clinical analysis purposes. Micro-scale enrichment columns (akin to Zip Tips™) may be needed to process, in both terms of analyte concentration and purity, the much smaller volume urine samples available/collected for large sample sets of clinical samples to be logistically (and economical) analysed by this proposed approach. Secondly, and as referred to above, a major criticism of MALDI TOF MS is that it is not quantifiable. That is the *y*-axis is a relative intensity within a profile and not directly proportional to the various amounts of given molecules present in the sample, *i.e.*, the molecules that ionize easily give more intense signals compared to molecules that might be more abundant but do not ionise easily, and therefore give weaker intensity signals. This reduces the value of MALDI TOF MS spectral data; but to partially overcome this we have adopted a normalisation approach in order to render peak intensities axis comparable between sample spectra. Thus, we transformed the *y*-axis values to a percentage of the spectral region being compared. It has yet to be seen if such a simple processing approach is sufficiently robust to be reproducible when comparing large numbers of samples in a clinical diagnostic situation.

There is some debate as to whether the carbohydrate composition of hCGβcf in pregnancy urine can be directly correlated to that of the parental hCGβ subunit. The results from this study are in line with literature that suggests that carbohydrate heterogeneity has been found in hCGβ in both normal and abnormal pregnancies and that this remains in the terminal urinary degradation product hCGβcf [13,28]. Other studies suggest that hCGβcf glycoforms are very different from that of the hCGβ subunit, proposing the presence of shortened asparagine-linked oligosaccharides on hCGβcf that had generally been metabolised to their pentasaccharide cores as well as smaller sugars [24,25,26,27]. One such study reported that 22%–44% of the hCGβcf failed to show binding ability to Concavalin A, which according to the authors is as a consequence of having no sugar molecules [27]. In the previous study, our group have shown that the hCGβcf glycosylated peptide β 6–40 is never completely trimmed of oligosaccharides and that there is only one non-glycosylated hCGβcf peptide, β 55–92 at *m*/*z* 4156.8 after reduction of hCGβcf with DTT [28]. This is also true for the samples in the current study. This discrepancy in the literature may be due to the difference in hCGβcf preparations; the source or the methods used for its purification and characterisation.

In our earlier study [28] hCGβcf was purified from pooled normal pregnancy urine samples and isolated by sequential size exclusion. In the present study the method of purification was ion exchange chromatography and in this case each sample; normal, molar and hyperemesis gravidarum was processed and analysed individually similar to that performed by Elliott *et al.* [13]. In fact some of the preparations used here were prepared alongside this study and as such can be compared directly to M4 and M2 hCG described therein. The largest oligosaccharides (*m*/*z* 2420.3 and 2598.4) detected in the pooled urine hCGβcf population previously were not detected in this cohort of patients in which the largest carbohydrate moiety was identified at *m*/*z* 2077.9. Previous studies in normal pregnancy suggest that by the 10th week of gestation some tri-antennary forms fall to less than 10% of total hCG, indicating that hCG tri-antennary glycoforms, including hyperglycosylated hCG are only seen in significant proportions earlier in pregnancy [13,32,33]. As the samples collected for this study were from the gestational period 7 to 13 weeks, it is possible that the proportion of hCGβcf composed of tri-antennary sugars in the urine samples from the weeks before gestation week 10 were diluted or cleared and in each of the samples this structure occurs in such low concentrations as to be undetectable by this technique.

Our previous study [28] proposed that there was a general absence of galactose with the pooled samples even though in one of the spectra a structure was observed that correlated to a single peak (*m*/*z* 1607.5) [28]. Peaks corresponding to this same glycoform (structure l, Figure 2) have been identified in the present study in all samples except M1βcf. Carbohydrate structures j, k, l, m and n (Figure 2) which have been attributed to peaks in the samples analysed for this study contain, in some isoforms, a galactose residue. In the literature the galactose content of hCGβcf has been reported differently. In some studies involving carbohydrate analysis after acid hydrolysis or the conversion of sugars to glycamines, small amounts of galactose has been detected [15,27]. In contrast to this, other groups have found hCGβcf *N*-linked sugars lacking galactose [24,26]. MALDI TOF MS analysis of the samples in this study has highlighted peaks which contribute significantly to the overall spectrum that cannot be correlated directly with currently identified carbohydrate moieties. It is tempting to speculate about the presence of additional hCGβcf peptide variants as has been suggested previously and their potential involvement in pregnancy and pregnancy associated disorders [14].

## 3. Materials and Methods

### 3.1. Biological Samples

Urine samples from five individual pregnancies were used in this study: two were complete molar pregnancies (M2βcf, M4βcf; *i.e.*, moles existed *in utero* when the urine sample was taken), one hyperemesis gravidarum (HGβcf), and two from apparently normal uncomplicated pregnancies (N1βcf, N2βcf). Because hCG reaches its highest levels in urine during the 10th week of pregnancy, all samples were obtained between the 7th and the 13th week of gestation, therefore allowing a 3-week window on either side of the hCG peak; 3 to 5 L of urine were collected continuously from each individual over several days. M2βcf and M4βcf were collected and stored (−80 °C) previously (and intact hCG extracted, the structure of which was reported earlier) [11]. The other samples were collected and purified at the University Of New Mexico School Of Medicine (Albuquerque, MN, USA) following full consent from pregnant women and ethical approval for the study was granted by the OB/GYN departmental research ethics committee.

### 3.2. Sample Treatments

Proteins were precipitated from urine, initially with acetone (acetone:urine = 2:1 (*v*:*v*)) (Merck, Nottingham, UK) overnight at 4 °C according to methods described previously [34]. The precipitate was collected by centrifugation, and re-dissolved in a minimum amount of distilled-deionized water and re-precipitated with ethanol (ethanol:sample = 9:1 (*v*:*v*)) (Merck) overnight at 4 °C. The resulting precipitate was collected by centrifugation, air-dried to remove excess ethanol, re-dissolved in a minimum amount of distilled-deionized water, and dialyzed against 0.05 M ammonium bicarbonate.

Samples M2βcf and M4βcf were initially fractionated by size exclusion chromatography on an S-200 Sephacryl column (Pharmacia, Piscataway, NJ, USA). The hCGβcf content of each fraction was then determined by specific immunoassay [35]. These samples were co-purified along with intact hCG, some of which were later characterized [13]. The hCGβcf fractions were lyophilized and stored at −80 °C.

All samples were then fractionated on a DEAE-Sepharose ion exchange column [36]. One hundred and thirty milliliter of DEAE-Sepharose CL-6B (Pharmacia, Piscataway, NJ, USA) was packed into an XK26 column (Pharmacia) (26 × 245 mm) at a flow rate of 1.5 mL/min. The void volume (*V*_0_) was calculated by detection of changes in salt concentration using silver nitrate precipitation, after the elution buffer was changed from 0.1 to 1 M ammonium bicarbonate (*V*_0_ = 144 mL). The column was then equilibrated with 2 L of 0.1 M ammonium bicarbonate buffer (pH 7.1) (Sigma-Aldrich, St. Louis, MO, USA) and kept at 4 °C at all times in order to prevent protein degradation by bacterial enzymes. Individual samples were loaded onto the column and the column was eluted with 200 mL of stepwise increases in ammonium bicarbonate buffer (pH 7.1) starting with 0.1 M at a flow rate of 1.5 mL/min, followed by 200 mL of 0.15 M, 200 mL of 0.2 M, 200 mL of 0.25 M and finally 200 mL of 1 M ammonium bicarbonate buffer. The eluent was collected as 9 mL fractions and the concentration of hCGβcf in each fraction was determined by enzyme-linked immunosorbent assay (ELISA).

### 3.3. hCGβcf Enzyme-Linked Immunosorbent Assay (ELISA)

The assay utilized a monoclonal antibody INN-hCG-106 against the β 11 epitope on hCGβcf as the capture antibody [37]. The S504 polyclonal antibody [38] was used as a primary detection antibody and a donkey-anti-sheep-HRP monoclonal (Jackson Immunoresearch Inc., West Grove, PA, USA) was used as a secondary detection antibody. All fractions with hCGβcf immunoreactivity were pooled and their hCGβcf levels were determined once again.

### 3.4. Matrix-Assisted Laser Desorption/Ionization Time-of-Flight Mass Spectrometry (MALDI TOF MS)

Post DEAE fractionation, samples were lyophilized against liquid nitrogen in order to remove buffers prior to mass spectrometric analysis. After two freeze-dry/rehydration cycles, the protein was re-dissolved in a minimum amount of distilled-deionized water.

### 3.5. Whole Molecule hCGβcf Analysis (Non-Reduced)

One micro-liter of sample was applied to a stainless steel MALDI TOF MS target and allowed to dry and crystallize at room temperature. 0.6 µL of sinapinic acid (20 mg/mL^−1^) (Sigma-Aldrich) in acetonitrile (Merck) and 0.1% trifluoroacetic acid (Merck) was applied on top of the sample and allowed to dry prior to mass spectrometric analysis.

### 3.6. Dithiothreitol (DTT)-Treated hCGβcf Analysis (Reduced)

Five micro-liters of neat sample was incubated with 5 µL of 100 mM DTT (Sigma-Aldrich) in 100 mM ammonium bicarbonate for 1 h at room temperature. Sample and matrix were then applied on the MALDI TOF MS target as described above.

A pulsed nitrogen laser (λ_max_ = 337 nm) was used to desorb ions from the sample, which were accelerated by a 20 kV electrical field down a 0.5 m linear tube and detected by a micro-channel plate detector. The detector was digitized at a sampling rate of 500 MHz. Spectra were generated by summing 20–30 laser shots by using a Finnigan LASERMAT 2000 instrument (Thermo-Finnigan, Waltham, MA, USA).

Mass calibration was assigned using horse heart cytochrome C (20 pmol/µL “on target”) as an external calibrant (two point calibration at [M + H]^+^ = 12,361 Da and [M + 2H]^2+^ = 6181 Da) for spectral analysis of whole hCGβcf. For spectra analysis of DTT-reduced hCGβcf, the non-glycosylated peptide of hCGβcf (β 55–92) was used as an internal calibrant (one point calibration at [M + H]^+^ = 4156.8 Da; calculated from its given primary sequence). A 0.5% error during peak mass allocation was allowed for, as this was typical in the linear mode for the MALDI instrument used.

### 3.7. Treatment of Spectra

In order to determine the masses of the carbohydrate moieties, a previously described method was used [28,39]: reduced hCGβcf spectra were calibrated by using the β 55–92 non-glycosylated peptide as described above. The inferred masses were determined by subtracting the mass of the glycosylated peptide of hCGβcf (β 6–40), which was calculated from its given primary sequence at a mass of 3752.4 Da. The carbohydrate content of each peak was then determined by sequential subtraction of the masses of individual sugar residues [28]. An error ≤0.25% was allowed between observed and predicted carbohydrate masses.

The percentage represented by each of the peaks in individual spectra was also calculated by using the following formula:

%Area = [(Peak height from baseline × Peak width at ½ height) × 100] ÷ Σ of Spectrum Peak Area

## 4. Conclusions

In conclusion, hCGβcf hyperglycosylation due to tri-antennary glycoforms was found to be the highest in the urine from women with molar and hyperemesis gravidarum pregnancies compared to the samples from normal pregnancy. Although such molecules are subject to metabolic processing, this supports previously published data from Elliott *et al.*, which has shown that hCG is *N*-linked hyperglycosylated to a greater extent in disease and abnormal pregnancy. Although a very high percentage of tri-antennary glycoforms were seen on the hCGβ subunit in abnormal pregnancy in that study [13], such distinct hyperglycosylation has not previously been seen as clearly in hCGβcf. The MALDI TOF MS technique described here, although not definitive is considerably simpler and faster than conventional approaches to glycostructural analysis and presents a potential novel approach to provide additional clinical information. Chromatographic purification prior to MALDI TOF MS analysis is still laborious; however, this may become unnecessary when coupled with affinity capture MALDI techniques as described by Neubert *et al.* [40] and in turn this may lead to more rapid analysis of multiple patients from spot urine samples.

The application of mass spectrometry in the analysis of glycosylation proteforms is developing rapidly. Glycomics, as demonstrated in Manfred Wuhrer’s recent review, is now entering the clinical diagnostic arena [41] and, as a result, international searchable databases specifically addressing glycosylation patterns are emerging [42].
